# CRISPR/Cascade 9-Mediated Genome Editing-Challenges and Opportunities

**DOI:** 10.3389/fgene.2018.00240

**Published:** 2018-07-05

**Authors:** Bhaskar Roy, Jing Zhao, Chao Yang, Wen Luo, Teng Xiong, Yong Li, Xiaodong Fang, Guanjun Gao, Chabungbam O. Singh, Lise Madsen, Yong Zhou, Karsten Kristiansen

**Affiliations:** ^1^BGI Genomics, BGI-Shenzhen, Shenzhen, China; ^2^Laboratory of Genomics and Molecular Biomedicine, Department of Biology, University of Copenhagen, Copenhagen, Denmark; ^3^School of Life Sciences, Tsinghua University, Beijing, China; ^4^Institute of Sericulture and Apiculture, College of Animal Sciences, Zhejiang University, Hangzhou, China; ^5^Institute of Marine Research, Bergen, Norway

**Keywords:** CRISPR, Cascade 9, genome editing, off-target effects, gene targeting, RNA guided system

## Abstract

Clustered Regularly Interspaced Palindromic Repeats (CRISPR) and Cascade 9 (also known as Cas9, CRISPR associated protein 9) confer protection against invading viruses or plasmids. The CRISPR/Cascade 9 system constitutes one of the most powerful genome technologies available to researchers today. So far, this technology has enabled efficient genome editing and modification in several model organisms and has successfully been used in biomedicine and biomedical engineering. However, challenges for efficient and safe genetic manipulation in several organisms persist. Here, we review functional approaches and future challenges associated with the use of the CRISPR/Cascade 9 genome editing system and discuss opportunities, ethical issues and future directions within this field.

## Introduction

**Figure 1:**
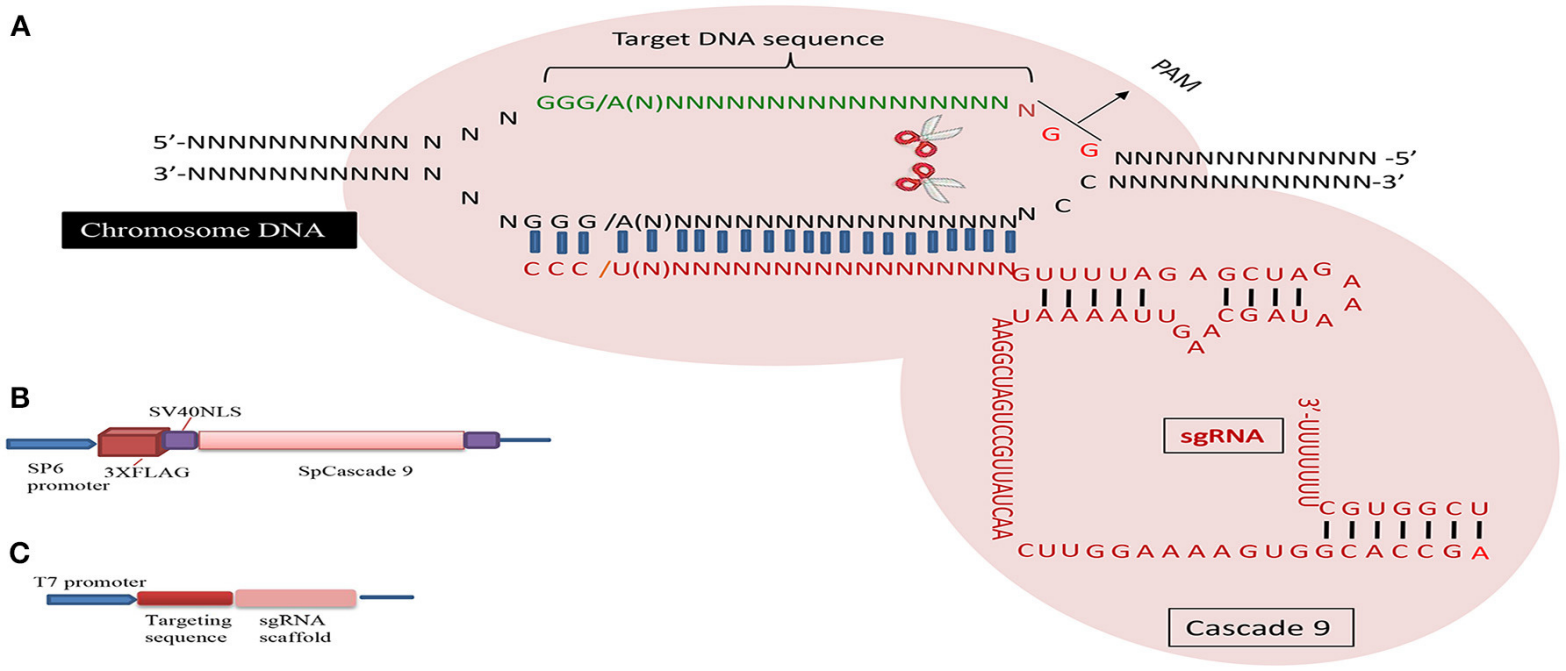
Schematic illustration of the CRISPR/Cascade 9 system and the genome editing process. **(A)** sgRNA is designed to target the genome with the standard sequence of 5′-GG(G/A)-N17/18-NGG-3′ at the 5′ of a PAM (NGG). **(B)** Map of the pSP6-2sNLS-SpCas9 vector. **(C)** Map of the pMD19-T sgRNA scaffold vector used to produce sgRNA driven by the T7 promoter.

**Figure 2:**
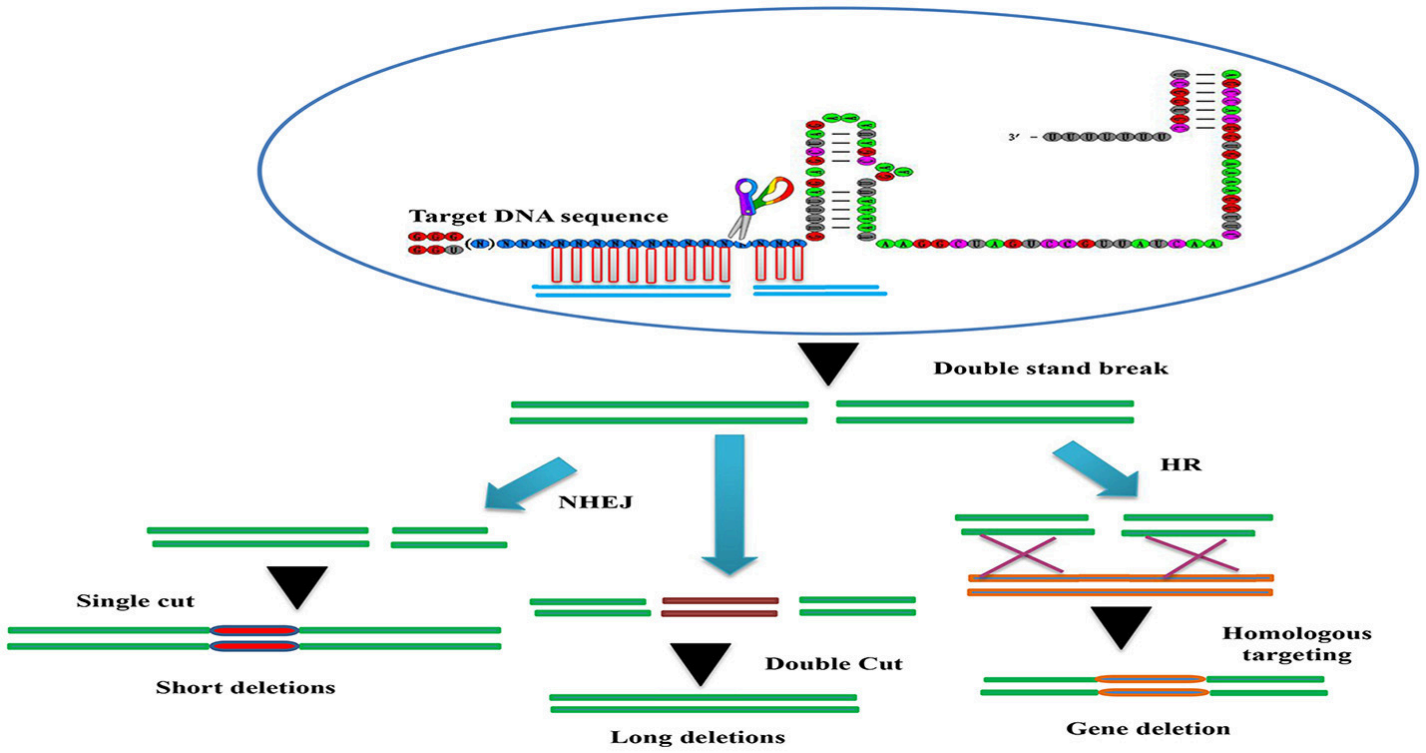
The basic working principle of the sgRNA-editing technology. Double strand break (DSB) repair can be used to target defined genomic modification. The double strand break (DSB) induced by the Cascade 9/sgRNA complex can be repaired through non-homologous end joining (NHEJ) or homologous recombination (HR). This can result in short insertions or deletions at the target site (left), deletions of larger genomic regions when two cuts are made (middle) or homologous repair with a desired template (brown). This can be used to alter the genome in a variety of different ways (bottom).

The CRISPR/Cascade 9 system is a RNA-guided system naturally used by archaea and bacteria for protection/immunity against invading viruses or plasmids and provides an interesting example of co-evolution between hosts and viruses. The simplicity of the CRISPR/Cascade 9 system has enabled the development of a reliable and efficient genome editing tool which has revolutionized the ability to manipulate genes in many different organisms (Peng et al., 2014). The CRISPR/Cascade 9 system comprises three components, small crRNAs (CRISPR RNAs), tracrRNA (auxiliary trans-activating crRNA), and the CRISPR associated protein 9 (Cascade 9). Cascade 9 is a RNA guided dsDNA nuclease which together with the crRNA/tracrRNA complex containing the target sequence of choice, termed the protospacer, is responsible for cleaving the targeted DNA strand using its HNH and RuvC nuclease domains. Cascade 9 also requires a small sequence downstream of the hybrid region called PAM (protospacer adjacent motif) that essentially behaves as a targeting component. The principle of the CRISPR/Cascade 9 system for targeted genome editing is illustrated in Figure 1. A synthetic fusion between crRNA and tracrRNA harboring a site-specific 20 bp single guide RNA (sgRNA) is constructed for the desired target site. The function of promoters directing the synthesis of sgRNAs is modulated by the number of guanidine residues at the 5′ end of the transcript. A single “G” is present in RNA polymerase III promoters, whereas two G's are found in SP6, T3, and T7 promoters. A separate vector drives expression of Cascade 9. The site-specific 20-nucleotides sequence of the sgRNA directs the Cascade 9 nuclease to its target. Double-strand breaks (DSBs) induced by Cascade 9 are re-ligated by the error-prone non-homologous end joining (NHEJ) pathway often resulting in deletions or insertions leading to loss of function (Figure 2). Structural and functional analyses have demonstrated that the PAM-binding domain of Cascade 9 interacts with the non-complementary strand GG dinucleotide via conserved arginine residues in the carboxy-terminal domain of Cascade 9, suggesting possibilities for overcoming the PAM-dependent limitation of targetable sequences (Anders et al., 2014). Accordingly, engineered SpCascade 9 and SaCascade 9 have recently been developed by utilizing bacterial selection-based directed evolution to recognize alternative PAMs (Kleinstiver et al., 2015). The SpCascade 9-VQR and SpCascade 9-EQR variants primarily recognize 5′-NGAN-3′ and 5′-NGNG-3′ PAMs, while the SpCascade 9-VRER variant is specific for the 5′-NGCG-3′ PMA motif, and using a structure-guided design strategy FnCascade 9 has been modified to recognize YG PAM sequences rather than NGG (Hirano et al., 2016).

Due to the simplicity and effectiveness of the CRISPR/Cascade 9 system, it is currently extensively used for genome editing in different organisms. Unlike protocols based on zinc-finger nucleases (ZNFs) and transcription activator-like effector nucleases (TALENs), protein engineering is not required, and it is possible to use sgRNAs targeting multiple sites. However, it should be noted that off-target modifications can be observed. The extent to which such off-target events occur seems to be correlated with the size and complexity of the targeted genome (Wei et al., 2014). Thus, reflecting genome sizes off-target events are more likely to occur in monocotyledonous plants than in dicotyledonous plants, and in vertebrates compared to invertebrates (Gregory et al., 2007) (Li and Du, 2014). In principle, the CRISPR/Cascade 9 system enables genome editing in embryos which, however, still is controversial. Furthermore, a number of technical issues still remain to be addressed (Chrenek et al., 2016), and specific deign for effective genome editing in embryos of different organisms may be required. For instance, the choice of delivery of the CRISPR/Cascade 9 system, electroporation, microinjection, and vector types used *in vitro* and *in vivo* remains challenging. In addition, toxicity of vectors and other components of CRISPR/Cascade 9 system also constitutes a challenge and needs to be addressed (Bassett et al., 2013). However, the successful correction of a gene in 42 out of 58 human embryos was recently reported (Ma et al., 2017).

Conventional gene disruption approaches such as gene deletion, gene insertion or frame-shift in coding sequences are all expensive and labor-intensive due to the inefficient homologous recombination-based protocols (Cong et al., 2013), and the use of alternative protocols using meganucleases has been limited due to the complex mechanism of genome editing associated with this approach (Silva et al., 2011). By contrast, already now the CRISPR/Cascade 9 system has been used for genome editing in a large variety of species as summarized in Table 1. Here we review different approaches and challenges associated with the use of the CRISPR/Cascade 9 system and discuss opportunities and future directions including ethical concerns.

**Table 1:** Examples of CRISPR/ Cascade 9-mediated genome editing in human cells and model organisms.

**Different approaches**	**Organisms**	**Genes**	**References**
Gene knockout	**INVERTERBRATES**		
	Drosophila	*Yellow, white, AGO1*	Bassett et al., 2013, 2014
	Silkworm	*BmWnt1, BmBLOS2* *Bm-ok, BmKMO, BmTH, and Bmtan*	Wang et al., 2013; Zhang et al., 2015 Wei et al., 2014
	*Caenorhabditis elegans*	*csr-1, mes-6, dpy-3, unc-1*	Cho et al., 2013b; Chen et al., 2015
	Yeast	*ADE2*	Giersch and Finnigan, 2017
	**VERTEBRATES**		
	Human	*H69, DMRT1, DMRT3, NF1, MED12*, *NF2, CUL3, TADA2B, TADA1, MAGEC2, S100A4, OCIAD1*	Shalem et al., 2014; Shetty and Inamdar, 2016; Tahara et al., 2016; Wang et al., 2016; Inui et al., 2017; Wills et al., 2017
	Zebrafish	*cyp19a1a, valopa, valopb*	Hang et al., 2016; Lau et al., 2016
	Mouse	*Rp9*	Lv et al., 2017
	Chicken	*Stra8, Myostatin*	Lee et al., 2017; Zhang et al., 2017
	Monkey	*Ppar-γ, Rag1*	Niu et al., 2014
	**PLANTS**		
	*Arabidopsis* Tobacco Sorghum and Rice	*OsSWEET14 and OsSWEET11, PDS3, TTG1, IAA2, CDK*	Jiang et al., 2013; Endo et al., 2015; Ryder et al., 2017; Tsutsui and Higashiyama, 2017
Gene knock-in	**INVERTERBRATES**		
	Drosophila	*nanos, yellow locus, white locus*	Xue et al., 2014; Port et al., 2015
	Silkworm	*Bmku70*	Ma et al., 2015
	*Caenorhabditis elegans*	*unc-119*	Zhao et al., 2014
	**VERTERBRATES**		
	Human	*DACT1, IFIT1and EGR1*	Zhang et al., 2006
	Zebrafish	*zebrafish th, tardbp, fus*	Armstrong et al., 2016
	Mouse	*Rosa26, KRAS, p53,LKB1*	Li et al., 2015
	Chicken	*yRad52*	Platt et al., 2014; Chu et al., 2016
	Pig	*COL1A*	Park et al., 2016; Wang et al., 2017a
	**PLANTS**		
	*Arabidopsis*	*PDS3, AtFLS2*	Li et al., 2014
	Tobacco	*No*	
	Rice	*WDV*	Wang et al., 2017
Gene Knockdown and silencing approaches	**INVERTERBRATES**		
	*Drosophila*	*roX1, roX2*,	Ghosh et al., 2016
	*Caenorhabditis elegans*	*TRHR-1*	
	Silkworm	No	Van Sinay et al., 2017
	**VERTERBRATES**		
	Human Zebrafish Mouse Chicken Pig	*EPHA1* *mmp21* *Nrl* No No	Cui et al., 2017 Guimier et al., 2015 Yu et al., 2017
Gene correction	**INVERTERBRATES**		
	*Drosophila*	No	
	*Caenorhabditis elegans*	No	
	Silkworm	No	
	**VERTERBRATES**		
	Human	*MYBPC3*	Ma et al., 2017
	Zebrafish	No	
	Mouse	*Hemophilia B, Pde6b*	
	Chicken	No	Huai et al., 2017
	Pig	No	
Conditional approaches	**INVERTERBRATES**		
	*Drosophila*	*bam, nos, cid, ms(3)k81, wg*	Port et al., 2014; Xue et al., 2014
	*Caenorhabditis elegans*	*dpy-5, lon-2, unc-76*	
	Silkworm	*No*	Shen et al., 2014
	**VERTERBRATES**		
	Human	*puroR, Ctnnb1*	Shen et al., 2014
	Zebrafish	*tyr, insra; insrb, ascl1a*	
	Mouse	*Mecp2, Ispd, Kras; p53;* *Lkb 1*	Yin L. et al., 2015
	Chicken	*No*	Yang et al., 2013; Lee and Lloyd, 2014; Platt et al., 2014
	Pig	*PFFs*	Liu et al., 2016

## Gene disruption approaches in organisms

### Knockout approaches

The use of the CRISPR/Cascade 9 system technology is rapidly increasing using different model systems and has allowed effective whole-genome screening for identification of therapeutic targets (see Table 1). Candidate gene knockout is a powerful approach as it may directly identify a phenotype resulting from the loss-of-function of the targeted gene. The CRISPR/Cascade 9-mediated knockout technology has been used in different genetic model organisms, including insects (Liu Y. et al., 2014; Xue et al., 2014), *Caenorhabditis elegans* (Liu P. et al., 2014) and humans (Tsai et al., 2014). Highly efficient mutagenesis of the *yellow* gene was achieved by injecting a sgRNA targeting an exon of the gene in *Drosophila* embryos (Bassett et al., 2013). As an example to illustrate the power of the CRISPR/Cascade 9 system, successful targeting of 14 distinct genomic loci in *Arabidopsis* with no detectable off-target events has been reported, also pointing to the CRISPR/Cascade 9 system as a powerful tool for agricultural purposes (Peterson et al., 2016).

Novel approaches for base-editing technology are essential for improving the efficiency of gene knockout or silencing. The recently developed WT-Cascade 9 -mediated gene knockout and CRISPR-STOP approaches, termed second generation genome editing tools, represent such two novel developments. The WT-Cascade 9 approach, which creates DSBs and indels at the target sites, allows exchange of a single specific base using the nickase activity of Cascade 9 to introduce C to T or A to G conversions at the specific target site (Gaudelli et al., 2017) (Komor et al., 2016). The CRISPR-STOP approach enables the insertion of early STOP codons in genes (Kuscu et al., 2017) with the codons CGA (Arg), CAG (Gln), and CAA (Gln) being effectively converted into stop codons with higher efficiency using the CRISPR-STOP approach than the WT-Cascade 9 knockout approach (Billon et al., 2017). Recently, Zuo et al. reported on a one–step generation of complete knockout of several genes in mouse and monkey models using multiple sgRN spaced 10–200 bp apart targeting a single key exon in each target gene. Phenotypic analysis of first generation mice revealed specific deletion of eight genes on the Y chromosome. This approach also resulted in highly-efficient gene knockout in monkeys (Zuo et al., 2017). Successful multiple gene knockout was further achieved by using a combination of dual sgRNAs in *C. elegans* where multiple sgRNA sequences were incorporated into a single CRISPR systems enabling selective editing and reducing off-target effect (Chen et al., 2015). Finally, in utero electroporation represents an interesting option for organ-specific gene knockout using the CRISPR/Cascade 9 system. As an example, Shinmyo et al. (2016) reported on a highly effective knockout of the *Satb2* gene in the mouse (Shinmyo et al., 2016).

### Knock-in approaches

Gene knock-in in different model organisms has traditionally been based on homologous recombination (HR) and NHEJ. Knock-in based on NHEJ has to some extent reduced time-consuming labor and has improved gene knock-in in cells and animals with low HR frequency (Yoshimi et al., 2016). However, double-strand DNA breaks (DSB) initiated by Cascade 9 are preferentially repaired by NHEJ, which is not appropriate for knocking-in genes. DSBs may also increase homology-directed repair (HDR)-dependent gene editing and knock-in, but several studies have recently shown that HDR-mediated gene knock-in in zebra fish (Auer et al., 2014; Irion et al., 2014) and Xenopus (Shi et al., 2015) was ineffective using the CRISPR/Cascade 9 system. Although the molecular mechanisms underlying DSB-induced mutagenesis have been intensely investigated, a number of questions remains concerning the use of homologous and micro-homologous sequences during template change in synthesis-dependent strand annealing (SDSA) and break-induced replication (BIR). Thus, it remains to be established to what extent the molecular mechanisms of SDSA and BIR have been maintained in different eukaryotes with different genome size or structure. A few studies using human cells have demonstrated that CRISPR/Cascade 9-NHEJ may result in effective DNA insertion and that the knock-in efficiency was improved compared with a HDR-based approach (He et al., 2016). Still, in a lung cancer mouse model, efficient knock-in of the *KRAS, p53, and LKB1* genes by CRISPR-based HDR has offered the ability to create defined modifications in genetic sequences associated with cancer (Platt et al., 2014). Interestingly, suppressing the NHEJ pathway or increasing the HDR pathway has been shown to enhance nuclease-mediated knock-in efficiency both *in vitro* and *in vivo*, and studies have further shown that small molecules such as Scr7, L755507 and resveratrol may enhance HDR-dependent knock-in efficiency of the CRISPR/Cascade 9 system. Thus, the supplementation with such small molecules may also be advantageous for production of genetically modified animals (Li et al., 2017). It should be mentioned that Suzuki and coworkers recently designed a micro-homology-mediated end joining (MMEJ)-assisted knock-in approach and a CRISPR-MMEJ-mediated tagging approach that have been applied in a variety of settings, ranging from cell lines such as HEK293T, HeLa, CHO-K1 to silkworm, zebrafish and frog (Sakuma et al., 2016). This vector delivery approach is efficient for gene editing, reaching 85% efficiency (Xiong et al., 2017). Finally, a recently described CRISPR-mediated epitope tagging approach holds promises for efficient gene knock-in (Nitika and Truman, 2017). So far, NHEJ-mediated repair of CRISPR/Cascade 9-mediated insertion of large DNA fragments has not been investigated in humans, and effective knock-in in human embryonic stem cell (ESCs) still constitutes a challenge. Experiments in model organisms that combine next-generation sequencing and genomic studies are clearly warranted to address remining problems associated with the use of the CRISPR/Cascade 9 system (Rodgers and McVey, 2016).

### Conditional approaches

**Figure 3:**
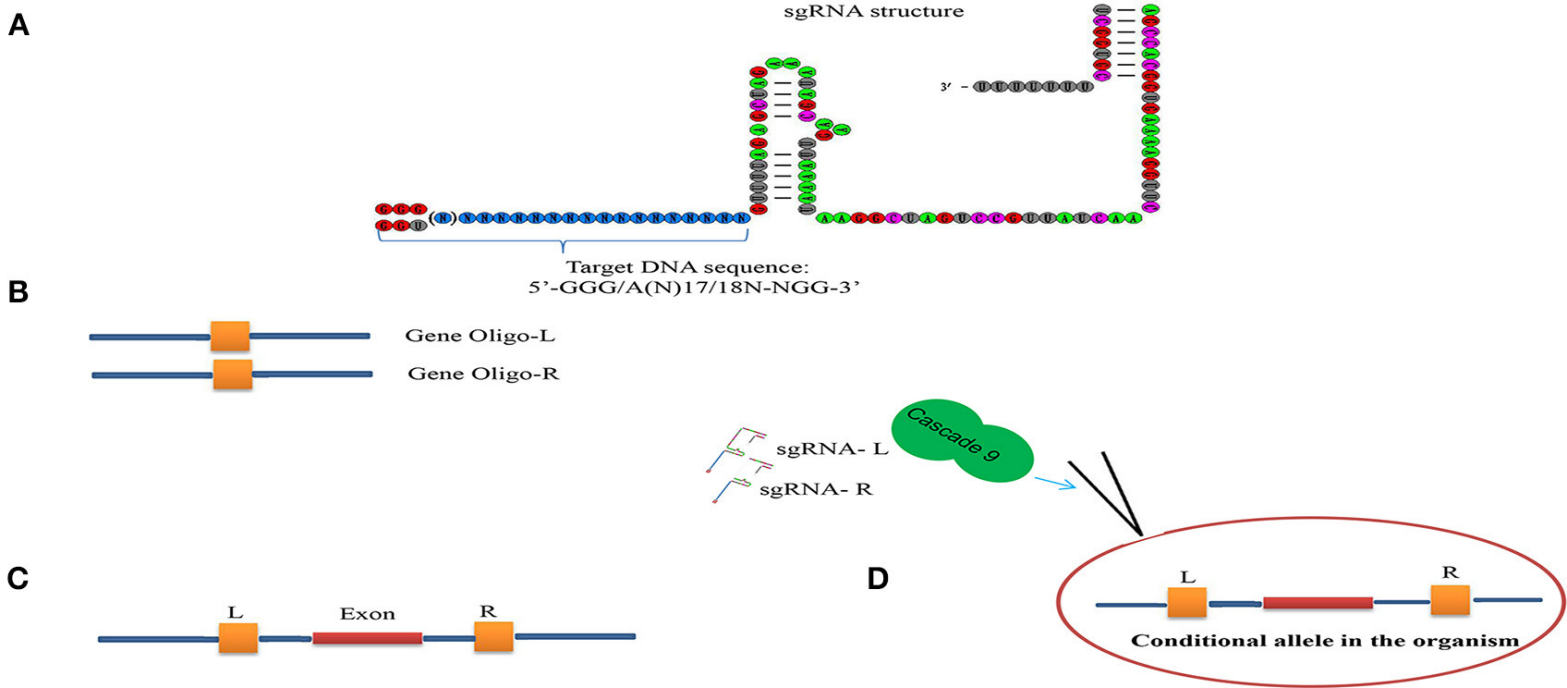
Scheme illustrating the sgRNA structure and mechanism of the target recognition. **(A)** The target DNA sequence is 5′-GG(G/A)-N17/18-NGG-3′ at the 5′ of a PAM (NGG). **(B)** Gene Oligo-L and Gen Oligo-R were deigned. **(C)** Gene exon was targeted by two sgRNA. **(D)** Cascade 9 and sgRNAs were microinjected into the organism.

**Figure 4:**
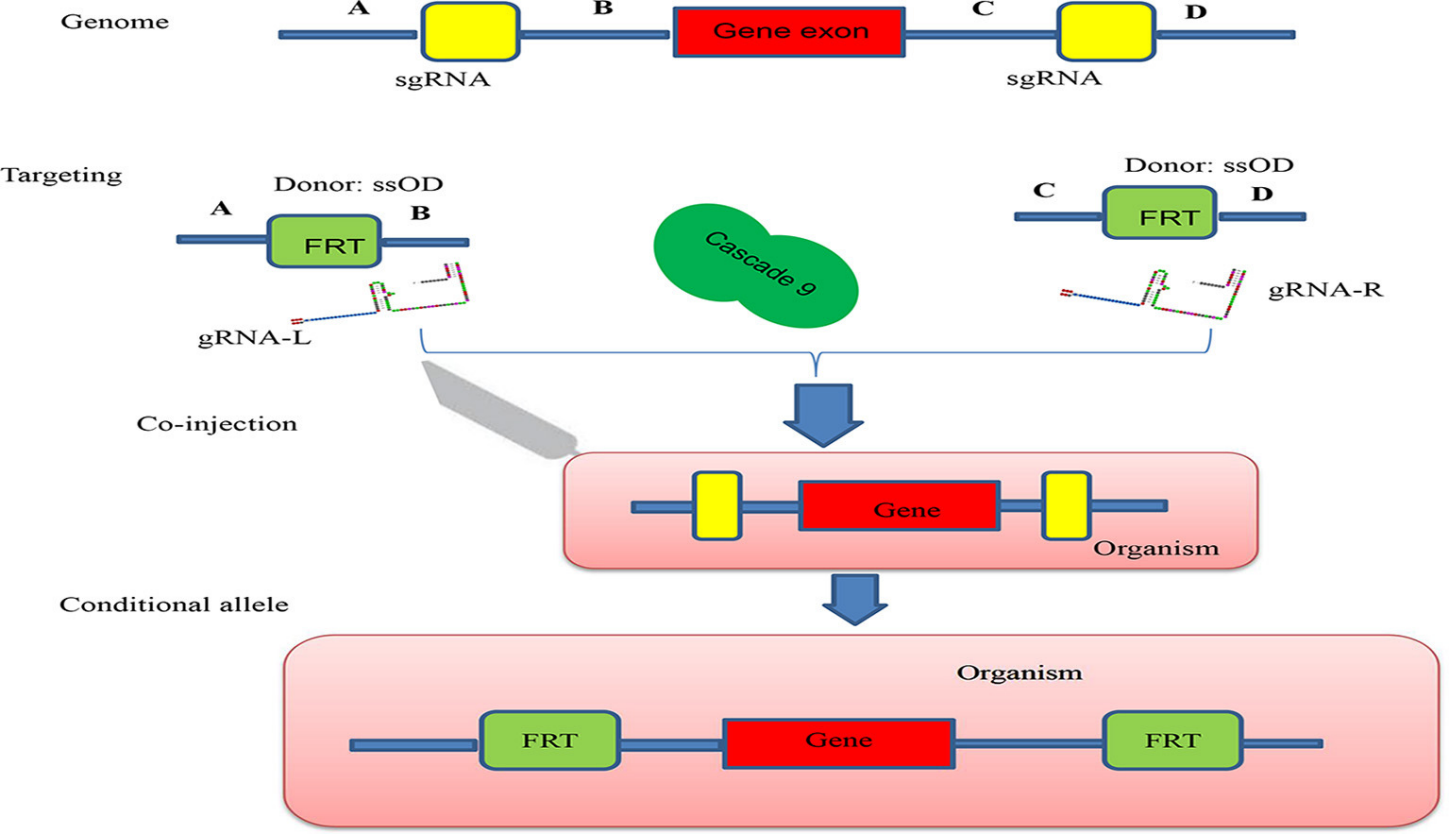
Schematic illustration of the principle behind generation of a conditional allele. A gene exon was targeted by two sgRNAs (sgRNA-L and sgRNA-R) which were designed to cut both ends of the gene exon, A single ssOD was designed to carry the two FRT sequences. All constructs and cascade 9 were microinjected into the organism.

Conditional knock-out (cKO) models circumvent a number of the problems related to the use of constitutive knock-out models such as embryonic lethality. cKO methods have been used widely for achieving tissue-specific gene deletion in the mouse (Kühn et al., 1995) and in the zebrafish. cKO models may further aid investigation and understanding of a number of human diseases. Conditional mutant models are thus becoming indispensable tools in the biomedical field (Rosenthal and Brown, 2007). Conditional approaches can overcome the limitations faced by simple knockout strategies using the CRISPR/Cascade 9 system for studies on embryogenesis as they allow temporal targeting of a specific tissue/organ at determined developmental stages and enable cell lineage tracing. As illustrated in Figure 3A, using the DNA sequence 5′-GG(G/A)-N17/18-NGG-3′ at the 5′ of a PAM (NGG) and designed Oligo-L and Oligo-R (Figure 3B), a specific gene exon can be targeted by two sgRNA following microinjected of Cascade 9 and the sgRNAs (Figures 3C,D). It has been demonstrated that the fusion of a *FKBP12*-derived destabilizing domain to Cascade 9 alters conditional Cascade 9 expression and the temporal control of gene editing in the presence of an *FKBP12* synthetic ligand allowing investigation of the interactions between functional genes (Figure 4). In conclusion, the CRISPR/Cascade 9 system has successfully been used to generate conditional knock-out mice, rats and *C. elegans* (Ma et al., 2014), demonstrating that the CRISPR/Cascade 9 is a powerful tool for investigating the relationship between genotype and phenotype in developmental biology.

### Gene correction approaches

Targeted gene correction may eventually be used for disease therapy including diseases such as muscular dystrophy, cystic fibrosis, hemophilia A and B, and Gauche disease. Previously, the cost of treatment, safety, and efficacy of gene editing has constituted huge obstacles, but some of these difficulties have now been circumvented by the development of CRISPR/Cascade 9 gene editing. For instance, Song et al. have successfully corrected mutations causing beta-thalassemia using iPSCs in combination with CRISPR/Cascade 9 without any observed off-target effects (Song et al., 2015). Further, the Cpf1 protein, a type V CRISPR effector, has been successfully used to induce mutations in the soybean genome (Fagerlund et al., 2015). Gene insertions or deletions can be introduced in cancerous cell using CRISPR/Cascade 9 technology. Thus, the CRISPR/Cascade 9 system may be used for insertion of inactivating indels in the oncogenic receptor tyrosine kinase *Erb2*, and the CRISPR/Cascade 9 technology has been applied in connection with lung cancer models and diseases including tyrosinemia and amyotrophic lateral sclerosis (Wang et al., 2017b; Yin et al., 2017b). In recent years, *Mycoplasma* and *Escherichia coli* genes have been edited *in vivo* in yeast cells using the CRISPR/Cascade 9 system (Tsarmpopoulos et al., 2016) and established standardized sequences of *S. cerevisiae* genes (selected from overexpression or gene knockdown) have been integrated into the yeast genome using the CRISPR/Cascade 9 system (Giersch and Finnigan, 2017). In the future, sgRNA may provide even better options for editing endogenous genomic sites and perform functional analysis of genes.

**Figure 5:**
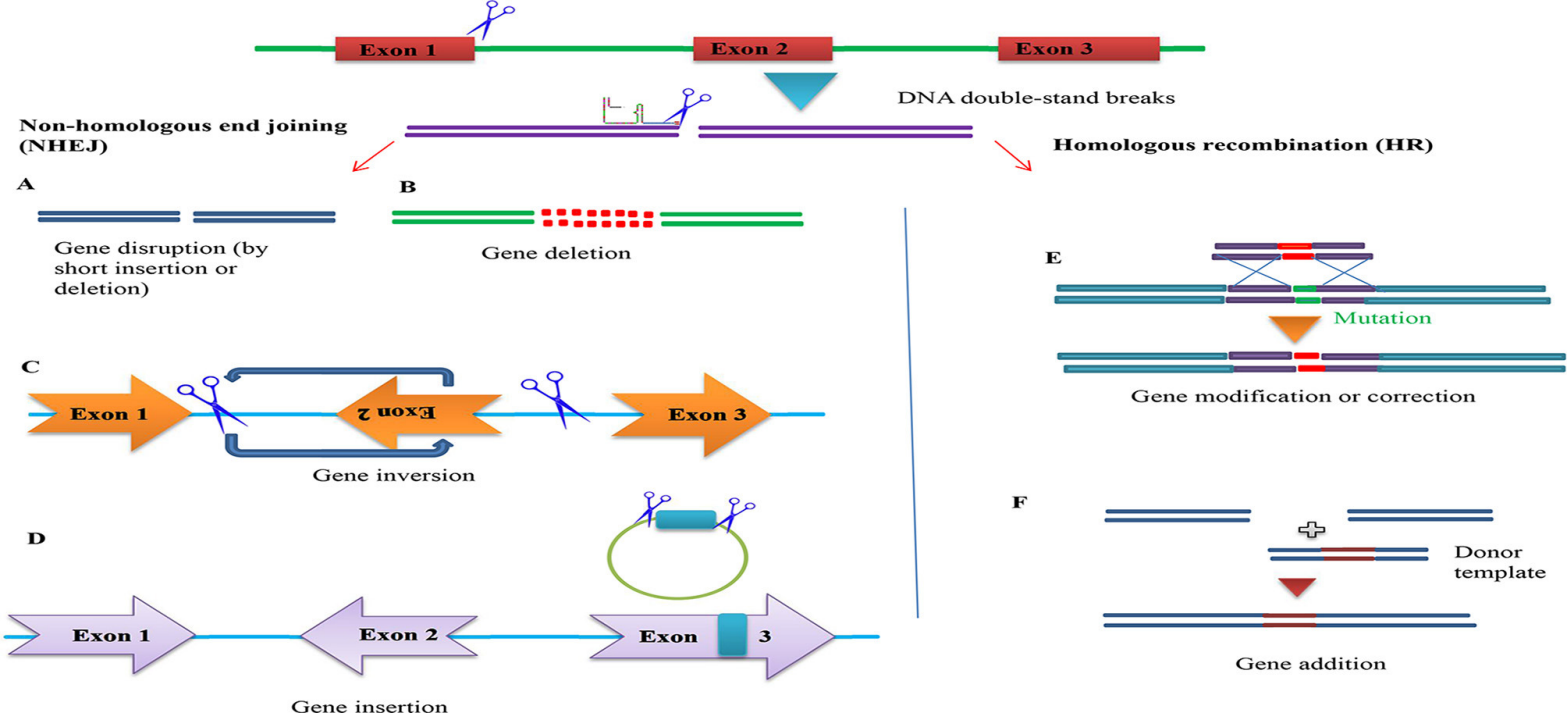
Overview of potential genome engineering outcomes using site-specific nucleases. (Left) Nuclease-induced DNA double-strand breaks (DSBs) can be repaired by homologous recombination (HR) or error-prone non-homologous end joining (NHEJ). In the absence of donor plasmid, NHEJ-mediated repair yields short insertion or deletion mutations at the target that cause gene disruption. DNA fragments up to 14 kb have been inserted via NHEJ-mediated ligation. Simultaneous induction of two DSBs can lead to deletions, inversions and translocations of the intervening segment **(A)**. Gene disruption by short insertion or deletion **(B)**. Gene deletion **(C)**. Gene inversion **(D)**. Gene insertion (Right) In the presence of a donor plasmid with extended homology, HR can lead to the introduction of single or multiple transgenes to correct or replace existing genes **(E)**. Gene addition **(F)**. Gene addition.

Gene correction approaches can be categorized into four types (1) endogenous gene disruption, (2) frame-shift to restore protein reading frame, (3) foreign sequence insertion using target-specific knock-in, and (4) mutated sequence substitution (Figure 5) (Lisa Li et al., 2014; Yin et al., 2017a). According to the Online Mendelian Inheritance in Man (OMIM) database, more than 8,000 inherited monogenic diseases arising from mutations in a single gene have been identified. However, an efficient drug therapy does not exist for the majority of these diseases. Therefore, gene therapy approaches by correcting the mutated loci using the CRISPR/Cascade 9 system represents a potential powerful tool for treatment. Yet increasing the efficiency of gene correction would be required for use in the biomedical field and off-target effects are of concern in relation to safety and side effects and this raises numerous questions in relation to ethics and safety (Yin H. et al., 2015).

## Gene knock-down and silencing approaches

The CRISPR/Cascade 9 technology has revolutionized molecular genetics and the technology may eventually be extensively used for gene functional studies. The efficiency of gene knock-down using the CRISPR/Cascade 9 system varies in relation to length and complementary to the crRNA sequence and the particular location of the gene (Gilbert et al., 2013). In the laboratory, the researchers face the choice between cutting-edge technologies and more established methods of RNA interference (RNAi)-mediated knock-down (Chang et al., 2016). Small interference RNA (siRNA), plasmid and virus-encoded short hairpin RNA are used in well-established procedures to knock-down target genes post-transcriptionally. By binding to the target mRNA, a siRNA will decrease stability and translation. The siRNA can be synthesized from a vector encoding a shRNA, an artificial RNA molecule containing a hairpin that is processed into the mature siRNA by the recipient cell enabling large-scale gene knockdown screening (Wang et al., 2016). Similarly, by producing a library of sgRNA targeting specific gene coding regions, the CRISPR/Cascade 9 systems can be used to screen for genes involved in molecular network. Recently, it was shown that Cas13a from *Leptotrichia wadei* enables targeted knock-down of RNA transcripts in bacteria, plant cells, and mammalian cells with an efficiency similar to that obtained by RNA interference, but with higher specificity. This approach can be applied for genome-wide knockdown screening, interrogation of lncRNA and nascent transcript function, including allele-specific knockdown, and RNA viral therapeutics (Abudayyeh et al., 2017)

The CRISPR/Cascade 9 system using catalytically inactive Cascade 9 is able to deliver 46–63% gene silencing in *HEK293* cells and exhibits even higher efficiency in *E. coli* (Qi et al., 2013). Although studies using CRISPR/Cascade 9 technology for complete gene knock-out may reveal more clear phenotypes and less false readouts compared with the variable knock-down of expression using RNAi, the efficiency of the CRISPR/Cascade 9 system is limited by the delivery of Cas9 and synthesis of guide RNA (gRNA) and further improvement is still needed.

## Off-target effects

Off-target effects represent one of the major challenges as Cascade 9 may recognize sequences with up to 5 mismatched bases. This means that off-target effects may occur to a higher rate than observed using other methods for genome editing. Some approaches to understand and overcome this limitation exist. Recent studies have revealed that DNA-RNA chimeric guides may provide a new strategy to reduce cost and off-target effects in human cells. Cpf1, a single RNA endonuclease utilizes a T-rich PAM on the 5′ side of the guide (Zetsche et al., 2016). Cpf1 originates from *Acidaminococcus* sp and a Cpf1-crRNA with an eight nucleotides DNA replacement at the 3′ end was produced. HEK293T cells were transfected with a plasmid expressing the Cpf1 protein followed by crRNAs after 24 h. This experiment showed that the Cpf1 crRNA with partial DNA replacement reduced off target effect in human cells (Yin et al., 2018). Novel ways to increase the targeting specificity and reduced off-target effects are actively pursued. Thus, it was reported that specific point mutations increased the specificity of SpCascade 9 (Kleinstiver et al., 2016). Similarly, CRISPR structure-guided rational designs have resulted in Cascade 9 variants with increased targeting specificity (Hsu et al., 2013). Studies aimed to re-engineer the Cascade 9 structure and alternative targeting approaches to reduce off-target effects are on-going. These efforts include deep sequencing of single targeted sequences in the genome to detect off-target loci, modification of guide RNA and Cascade 9 (Cho et al., 2013a) as well as correcting off-target mutations by back crossing to the wild-type (Bassett and Liu, 2014). Further, studies have shown that nick Cascade 9 can improve the specificity of target recognition in mammalian cell (Fujii et al., 2014) and a double-nicking strategy can reduce off-target effects of Cascade 9 (Ran et al., 2014). Finally, one study reported that paired Cascade 9 nickases, composed of D10A Cascade 9 and guide RNA, which produce two single-strand breaks (SSBs) or nicks in the DNA strands, were highly specific in human cells, avoiding off-target mutations without sacrificing genome-editing efficiency demonstrating that such strategy could be effective for genome editing without significant off-target effects (Cho et al., 2014).

In conclusion, reducing off-target effects may expand the applications of this gene-editing technology (Kleinstiver et al., 2016). To achieve this goal, more studies to understand the off-target mechanism and better design of sgRNA are important and deep sequencing analysis or high-throughput sequencing of numerous sgRNAs may provide important information regarding potential off-targets associated with the CRISPR/Cascade 9 system.

## Conclusions and perspectives

Despite the success achieved by the use of ZFNs, TALENs, and the RNA-guided Cascade 9 system, important challenges related to the effectiveness, specificity, and safety of the CRISPR/Cascade 9 system remain. In addition, the optimal RNA scaffold of CRISPR/Cascade 9 for application in several eukaryotic systems is not known. It also remains to comprehensively evaluate off-targets of Cascade 9 nucleases in the genome of interest. In this context, construction of novel versions of Cascade 9 or sgRNAs/crRNAs may improve the on- and off-target ratio. A system identified in the bacterium *Neisseria meningitides* and uses a longer recognition sequence was recently described (Zhang et al., 2013). Finally, it has been suggested that lower concentrations of CRISPR/Cascade 9 reagents in cell diminished cleaving at off-target sites compared to cleaving at on-target sites (Pattanayak et al., 2013). To transform the future of genetic biology, an in-depth analysis of ZFN, TALENs, or CRISPR/Cascade 9 systems based on visualization, detection and purification of protein is needed, but so far the CRISPR/Cascade 9 system seems to be the system of choice. Thus, detailed studies of gene function by using the CRISPR/Cascade 9 system have provided novel insight indicating that the CRISPR/Cascade 9 system has the potential to become the key genome editing tool in modern biology (Table 1). However, further investigations into the CRISPR/Cascade 9 system are clearly required to determine its effectiveness in generating a versatile and heritable modification of target genes, specifically in animals and plants, and more knowledge clearly is needed, and optimization for different cell types and organisms will be required prior to therapeutically and clinical applications.

During transfection or micro-injection with standard viral vectors some cells or tissues could be refractory. Hence, it is a challenge to develop methods securing specific and effective expression of the Cascade 9 nuclease and/or the sgRNAs for each cell type or developmental stage. Additionally, ensuring that a single vector efficiently expresses numerous gRNAs would expand the use of the CRISPR/Cascade 9 systems. Altogether, such improvements would be very important in relation to research and therapeutic applications. Improving methods to specifically change the structure or function of a given gene will allow reverse genetics approaches for any cell type or organism of interest. Still, the levels of Cascade 9 off-target effects remain a problem and the attempts to improve specificity are of importance. The recently developed catalytically dead Cascade 9 (dCas9) 9-targeted somatic hypermutation (a strategy known as CRISPR-X) technology for protein engineering enables specific mutation of endogenous targets with limited off-target damage and is a potentially powerful new approach to investigate the relationship between drug and protein (Hess et al., 2016).

The CRISPR/Cascade 9 technique was recently used to remove porcine endogenous retroviruses (PERVs) from the porcine genome, an achievement of importance in relation to future organ transplantation from pigs to human. However, more work regarding safety of cross-species viral transmission is required (Niu et al., 2017). One interesting CRISPR application relates to “gene drive” that can target whole population or a species. Here it has been demonstrated that a gene allele that confers a parasite-resistant phenotype in mosquitos can rapidly spread into the population in a non-Mendelian fashion (Gantz et al., 2015; Hammond et al., 2016).

Genome editing technology has emerged as a promising field in relation to medical applications, but guidelines for designing preclinical trials studies and ethical issues surrounding genome editing and genomic analysis at a population level remain to be established. Thus, the ethical issues regarding the application of the CRISPR/Cascade 9 system in relation to gene therapy are huge (Cyranoski, 2015; Lanphier et al., 2015; Ma et al., 2017). Ethical issues comprise problems of safety, setups in clinical trials, and the criteria for including patients for research and treatment. Key ethical questions in relation to gene therapy in clinical trials also include decisions on criteria for when to implement a new concept in gene therapy where the long term consequences are unknown. Such ethical issues are of paramount importance and the involvement of and accept from the public before instigating such radical novel modalities for treatment of human diseases represent a genuine challenge.

Since the development of the CRISPR/Cascade 9 system, novel opportunities in the field of biomedical research have emerged. However, the effectiveness, accuracy, resolution, and genetic mutation variation still depend on several factors including sgRNA design, DSB introduced, and quantity of nuclease. The safety of the Cascade 9 system for introduction of heritable modifications of specific genes in plants and animals remains to be fully elucidated. Especially, the off-target effects of this system remain to be determined on a large genome-wide scale. Attention clearly needs to be devoted to future applications such as gene therapy and drug screening in the medical field. While the CRISPR/Cascade 9 technology has proven its applicability within numerous lines of basic research, ethical issues as discussed above are needed to be addressed and internationally accepted guidelines need to be adopted for the further use of this technology for genome editing in humans.

## Author contributions

XF, BR decided on the content and structure of the first version of the manuscript. JZ, CY, WL, TX, YL collected the information. BR drafted the first version of the manuscript while GG, CS, YZ, LM, and KK extensively restructured and revised the manuscript. All authors have read and approved the final version of the manuscript for publication.

### Conflict of interest statement

The authors declare that the research was conducted in the absence of any commercial or financial relationships that could be construed as a potential conflict of interest.

## References

[B1] Abudayyeh O. O. Gootenberg J. S. Essletzbichler P. Han S. Joung J. Belanto J. J. (2017). RNA targeting with CRISPR-Cas13a. Nature 550, 280–284. 10.1038/nature24049 28976959 PMC5706658

[B2] Anders C. Niewoehner O. Duerst A. Jinek M. (2014). Structural basis of PAM-dependent target DNA recognition by the Cas9 endonuclease. Nature 513, 569–573. 10.1038/nature13579 25079318 PMC4176945

[B3] Armstrong G. A. B. Liao M. You Z. Lissouba A. Chen B. E. Drapeau P. (2016). Homology directed knockin of point mutations in the zebrafish tardbp and fus genes in ALS using the CRISPR/Cas9 system. PLoS ONE 11:e0150188. 10.1371/journal.pone.0150188 26930076 PMC4773037

[B4] Auer T. O. Duroure K. De Cian A. Concordet J. P. Del Bene F. (2014). Highly efficient CRISPR/Cas9-mediated knock-in in zebrafish by homology-independent DNA repair. Genome Res. 24, 142–153. 10.1101/gr.161638.113 24179142 PMC3875856

[B5] Bassett A. Liu J. L. (2014). CRISPR/Cas9 mediated genome engineering in Drosophila. Methods 69, 128–136. 10.1016/j.ymeth.2014.02.019 24576617

[B6] Bassett A. R. Tibbit C. Ponting C. P. Liu J. L. (2013). Highly efficient targeted mutagenesis of Drosophila with the CRISPR/Cas9 System. Cell Rep. 4, 220–228. 10.1016/j.celrep.2013.06.020 23827738 PMC3714591

[B7] Bassett A. R. Tibbit C. Ponting C. P. Liu J. L. (2014). Mutagenesis and homologous recombination in Drosophila cell lines using CRISPR/Cas9. Biol. Open 3, 42–49. 10.1242/bio.20137120 24326186 PMC3892159

[B8] Billon P. Bryant E. E. Joseph S. A. Nambiar T. S. Hayward S. B. Rothstein R.. (2017). CRISPR-mediated base editing enables efficient disruption of eukaryotic genes through induction of STOP codons. Mol. Cell 67, 1068–1079.e4. 10.1016/j.molcel.2017.08.008 28890334 PMC5610906

[B9] Chang H. Yi B. Ma R. Zhang X. Zhao H. Xi Y. (2016). CRISPR/cas9, a novel genomic tool to knock down microRNA *in vitro* and *in vivo*. Sci. Rep. 6, 22312. 10.1038/srep22312 26924382 PMC4770416

[B10] Chen X. Li M. Feng X. Guang S. (2015). Targeted chromosomal translocations and essential gene knockout using CRISPR/Cas9 technology in caenorhabditis elegans. Genetics 201, 1295–1306. 10.1534/genetics.115.181883 26482793 PMC4676527

[B11] Cho S. W. Kim S. Kim J. M. Kim J. S. (2013a). Targeted genome engineering in human cells with the Cas9 RNA-guided endonuclease. Nat. Biotechnol. 31, 230–232. 10.1038/nbt.2507 23360966

[B12] Cho S. W. Kim S. Kim Y. Kweon J. Kim H. S. Bae S.. (2014). Analysis of off-target effects of CRISPR/Cas-derived RNA-guided endonucleases and nickases. Genome Res. 24, 132–141. 10.1101/gr.162339.113 24253446 PMC3875854

[B13] Cho S. W. Lee J. Carroll D. Kim J. S. Lee J. (2013b). Heritable gene knockout in *Caenorhabditis elegans* by direct injection of Cas9-sgRNA ribonucleoproteins. Genetics 195, 1177–1180. 10.1534/genetics.113.155853 23979576 PMC3813847

[B14] Chrenek M. A. Nickerson J. M. Boatright J. H. (2016). Clustered regularly interspaced short palindromic repeats: challenges in treating retinal disease. Asia Pac. J. Ophthalmol. 5, 304–308. 10.1097/APO.0000000000000225 27488072 PMC4975549

[B15] Chu V. T. Weber T. Graf R. Sommermann T. Petsch K. Sack U.. (2016). Efficient generation of Rosa26 knock-in mice using CRISPR/Cas9 in C57BL/6 zygotes. BMC Biotechnol. 16:4. 10.1186/s12896-016-0234-4 26772810 PMC4715285

[B16] Cong L. Ran F. A. Cox D. Lin S. Barretto R. Hsu P. D.. (2013). Multiplex genome engineering using CRISPR/VCas systems. Science 339, 819–823. 10.1126/science.1231143 23287718 PMC3795411

[B17] Cui Y. Wu B. O. Flamini V. Evans B. A. J. Zhou D. Jiang W. G. (2017). Knockdown of EPHA1 using CRISPR/CAS9 suppresses aggressive properties of ovarian cancer cells. Anticancer Res. 37, 4415–4424. 10.21873/anticanres.11836 28739735

[B18] Cyranoski D. (2015). Embryo editing divides scientists. Nature 519, 272. 10.1038/519272a 25788074

[B19] Endo M. Mikami M. Toki S. (2015). Multigene knockout utilizing off-target mutations of the CRISPR/cas9 system in rice. Plant Cell Physiol. 56, 41–47. 10.1093/pcp/pcu154 25392068 PMC4301742

[B20] Fagerlund R. D. Staals R. H. J. Fineran P. C. (2015). The Cpf1 CRISPR-Cas protein expands genome-editing tools. Genome Biol. 16, 15–17. 10.1186/s13059-015-0824-9 26578176 PMC4647450

[B21] Fujii W. Onuma A. Sugiura K. Naito K. (2014). Efficient generation of genome-modified mice via offset-nicking by CRISPR/Cas system. Biochem. Biophys. Res. Commun. 445, 791–794. 10.1016/j.bbrc.2014.01.141 24491566

[B22] Gantz V. M. Jasinskiene N. Tatarenkova O. Fazekas A. Macias V. M. Bier E.. (2015). Highly efficient Cas9-mediated gene drive for population modification of the malaria vector mosquito *Anopheles stephensi*. Proc. Natl. Acad. Sci. U.S.A. 112, E6736–E6743. 10.1073/pnas.1521077112 26598698 PMC4679060

[B23] Gaudelli N. M. Komor A. C. Rees H. A. Packer M. S. Badran A. H. Bryson D. I.. (2017). Programmable base editing of A • T to G • C in genomic DNA without DNA cleavage. Nat. Publ. Gr. 551, 464–471. 10.1038/nature24644 29160308 PMC5726555

[B24] Ghosh S. Tibbit C. Liu J. L. (2016). Effective knockdown of Drosophila long non-coding RNAs by CRISPR interference. Nucleic Acids Res. 44:e84. 10.1093/nar/gkw063 26850642 PMC4872081

[B25] Giersch R. M. Finnigan G. C. (2017). Yeast still a beast: diverse applications of CRISPR/CAS editing technology in *S. cerevisiae*. Yale J. Biol. Med. 90, 643–651. 29259528 PMC5733842

[B26] Gilbert L. A. Larson M. H. Morsut L. Liu Z. Brar G. A. Torres S. E.. (2013). XCRISPR-mediated modular RNA-guided regulation of transcription in eukaryotes. Cell 154, 442–451. 10.1016/j.cell.2013.06.044 23849981 PMC3770145

[B27] Gregory T. R. Nicol J. A. Tamm H. Kullman B. Kullman K. Leitch I. J.. (2007). Eukaryotic genome size databases. Nucleic Acids Res. 35, 332–338. 10.1093/nar/gkl828 17090588 PMC1669731

[B28] Guimier A. Gabriel G. C. Bajolle F. Tsang M. Liu H. Noll A.. (2015). MMP21 is mutated in human heterotaxy and is required for normal left-right asymmetry in vertebrates. Nat. Genet. 47, 1260–1263. 10.1038/ng.3376 26437028 PMC5620017

[B29] Hammond A. Galizi R. Kyrou K. Simoni A. Siniscalchi C. Katsanos D.. (2016). A CRISPR-Cas9 gene drive system targeting female reproduction in the malaria mosquito vector *Anopheles gambiae*. Nat. Biotechnol. 34, 78–83. 10.1038/nbt.3439 26641531 PMC4913862

[B30] Hang C. Y. Moriya S. Ogawa S. Parhar I. S. (2016). Deep brain photoreceptor (val-opsin) gene knockout using CRISPR/Cas affects chorion formation and embryonic hatching in the zebrafish. PLoS ONE 11:e0165535. 10.1371/journal.pone.0165535 27792783 PMC5085036

[B31] He X. Tan C. Wang F. Wang Y. Zhou R. Cui D.. (2016). Knock-in of large reporter genes in human cells via CRISPR/Cas9-induced homology-dependent and independent DNA repair. Nucleic Acids Res. 44:e85. 10.1093/nar/gkw064 26850641 PMC4872082

[B32] Hess G. T. Frésard L. Han K. Lee C. H. Li A. Cimprich K. A.. (2016). Directed evolution using dCas9-targeted somatic hypermutation in mammalian cells. Nat. Methods 13, 1036–1042. 10.1038/nmeth.4038 27798611 PMC5557288

[B33] Hirano H. Gootenberg J. S. Horii T. Abudayyeh O. O. Kimura M. Hsu P. D.. (2016). Structure and engineering of Francisella novicida Cas9. Cell 164, 950–961. 10.1016/j.cell.2016.01.039 26875867 PMC4899972

[B34] Hsu P. D. Scott D. A. Weinstein J. A. Ran F. A. Konermann S. Agarwala V. (2013). Rationally engineered Cas9 nulceases with improved specificity. Nat. Biotechnol. 31, 827–832. 10.1038/nbt.2647 23873081 PMC3969858

[B35] Huai C. Jia C. Sun R. Xu P. Min T. Wang Q.. (2017). CRISPR/Cas9-mediated somatic and germline gene correction to restore hemostasis in hemophilia B mice. Hum. Genet. 136, 875–883. 10.1007/s00439-017-1801-z 28508290

[B36] Inui M. Tamano M. Kato T. Takada S. (2017). CRISPR/Cas9-mediated simultaneous knockout of Dmrt1 and Dmrt3 does not recapitulate the 46,XY gonadal dysgenesis observed in 9p24.3 deletion patients. Biochem. Biophys. Rep. 9, 238–244. 10.1016/j.bbrep.2017.01.001 28956011 PMC5614593

[B37] Irion U. Krauss J. Nüsslein-Volhard C. (2014). Precise and efficient genome editing in zebrafish using the CRISPR/Cas9 system. Development 141, 4827–4830. 10.1242/dev.115584 25411213 PMC4299274

[B38] Jiang W. Zhou H. Bi H. Fromm M. Yang B. Weeks D. P. (2013). Demonstration of CRISPR/Cas9/sgRNA-mediated targeted gene modification in Arabidopsis, tobacco, sorghum and rice. Nucleic Acids Res. 41, 1–12. 10.1093/nar/gkt780 23999092 PMC3814374

[B39] Kleinstiver B. P. Pattanayak V. Prew M. S. Tsai S. Q. Nguyen N. T. Zheng Z.. (2016). High-fidelity CRISPR–Cas9 nucleases with no detectable genome-wide off-target effects. Nature 529, 490–495. 10.1038/nature16526 26735016 PMC4851738

[B40] Kleinstiver B. P. Prew M. S. Tsai S. Q. Topkar V. V. Nguyen N. T. Zheng Z.. (2015). Engineered CRISPR-Cas9 nucleases with altered PAM specificities. Nature 523, 481–485. 10.1038/nature14592 26098369 PMC4540238

[B41] Komor A. C. Kim Y. B. Packer M. S. Zuris J. A. Liu D. R. (2016). Programmable editing of a target base in genomic DNA without double-stranded DNA cleavage. Nature 533, 420–424. 10.1038/nature17946 27096365 PMC4873371

[B42] Kühn R. Schwenk F. Aguet M. Rajewsky K. (1995). Inducible gene targeting in mice. Science 269, 1427–1429. 10.1126/science.7660125 7660125

[B43] Kuscu C. Parlak M. Tufan T. Yang J. Szlachta K. Wei X.. (2017). CRISPR-STOP: Gene silencing through base-editing-induced nonsense mutations. Nat. Methods 14, 710–712. 10.1038/nmeth.4327 28581493

[B44] Lanphier E. Urnov F. Haecker S. E. Werner M. Smolenski J. (2015). Don't edit the human germ line. Nature 519, 410–411. 10.1038/519410a 25810189

[B45] Lau E. S. Zhang Z. Qin M. Ge W. (2016). Knockout of zebrafish ovarian aromatase gene (cyp19a1a) by TALEN and CRISPR/Cas9 leads to all-male offspring due to failed ovarian differentiation. Sci. Rep. 6:37357. 10.1038/srep37357 27876832 PMC5120357

[B46] Lee A. Y. Lloyd K. C. (2014). Conditional targeting of Ispd using paired Cas9 nickase and a single DNA template in mice. FEBS Open Biol. 4, 637–642. 10.1016/j.fob.2014.06.007 25161872 PMC4141200

[B47] Lee J. H. Kim S. W. Park T. S. (2017). Myostatin gene knockout mediated by Cas9-D10A nickase in chicken DF1 cells without off-target effect. Asian Austral. J. Anim. Sci. 30, 743–748. 10.5713/ajas.16.0695 27764916 PMC5411835

[B48] Li G. Zhang X. Zhong C. Mo J. Quan R. Yang J.. (2017). Small molecules enhance CRISPR/Cas9-mediated homology-directed genome editing in primary cells. Sci. Rep. 7, 1–11. 10.1038/s41598-017-09306-x 28827551 PMC5566437

[B49] Li J. Aach J. Norville J. E. McCormack M. Bush J. Church G. M.. (2014). Multiplex and homologous recombination-mediated genome editing in Arabidopsis and Nicotiana benthamiana using guide RNA and Cas9. Nat. Biotechnol. 31, 688–691. 10.1038/nbt.2654 23929339 PMC4078740

[B50] Li J. Zhang B. Ren Y. Gu S. Xiang Y. Du J. (2015). Intron targeting-mediated and endogenous gene integrity-maintaining knockin in zebrafish using the CRISPR/Cas9 system. Cell Res. 25, 634–637. 10.1038/cr.2015.43 25849248 PMC4423083

[B51] Li X. Q. Du D. (2014). Variation, evolution, and correlation analysis of C+G content and genome or chromosome size in different kingdoms and phyla. PLoS ONE 9:e88339. 10.1371/journal.pone.0088339 24551092 PMC3923770

[B52] Lisa Li H. Nakano T. Hotta A. (2014). Genetic correction using engineered nucleases for gene therapy applications. Dev. Growth Differ. 56, 63–77. 10.1111/dgd.12107 24329887

[B53] Liu G. Liu K. Wei H. Li L. Zhang S. (2016). Generation of porcine fetal fibroblasts expressing the tetracycline-inducible Cas9 gene by somatic cell nuclear transfer. Mol. Med. Rep. 14, 2527–2533. 10.3892/mmr.2016.5530 27430306 PMC4991725

[B54] Liu P. Long L. Xiong K. Yu B. Chang N. Xiong J.-W.. (2014). Heritable/conditional genome editing in *C. elegans* using a CRISPR-Cas9 feeding system. Cell Res. 24, 886–889. 10.1038/cr.2014.73 24874953 PMC4085767

[B55] Liu Y. Ma S. Wang X. Chang J. Gao J. Shi R.. (2014). Highly efficient multiplex targeted mutagenesis and genomic structure variation in *Bombyx mori* cells using CRISPR/Cas9. Insect Biochem. Mol. Biol. 49, 35–42. 10.1016/j.ibmb.2014.03.010 24698835

[B56] Lv J.-N. Zhou G.-H. Chen X. Chen H. Wu K.-C. Xiang L.. (2017). Targeted RP9 ablation and mutagenesis in mouse photoreceptor cells by CRISPR-Cas9. Sci. Rep. 7:43062. 10.1038/srep43062 28216641 PMC5317003

[B57] Ma H. Marti-Gutierrez N. Park S.-W. Wu J. Lee Y. Suzuki K.. (2017). Correction of a pathogenic gene mutation in human embryos. Nature 548, 413–419. 10.1038/nature23305 28783728

[B58] Ma S. Chang J. Wang X. Liu Y. Zhang J. Lu W.. (2015). CRISPR/Cas9 mediated multiplex genome editing and heritable mutagenesis of BmKu70 in *Bombyx mori*. Sci. Rep. 4:4489. 10.1038/srep04489 24671069 PMC3967148

[B59] Ma Y. Zhang X. Shen B. Lu Y. Chen W. Ma J.. (2014). Generating rats with conditional alleles using CRISPR/Cas9. Cell Res. 24, 122–125. 10.1038/cr.2013.157 24296780 PMC3879705

[B60] Niu D. Wei H. Lin L. George H. Wang T. Lee I.-H.. (2017). Inactivation of porcine endogenous retrovirus in pigs using CRISPR-Cas9. Science 357, 1303–1307. 10.1126/science.aan4187 28798043 PMC5813284

[B61] Niu Y. Shen B. Cui Y. Chen Y. Wang J. Wang L.. (2014). Generation of gene-modified cynomolgus monkey via Cas9/RNA-mediated gene targeting in one-cell embryos. Cell 156, 836–843. 10.1016/j.cell.2014.01.027 24486104

[B62] Nitika Truman A. W. (2017). Endogenous epitope tagging of heat shock protein 70 isoform Hsc70 using CRISPR/Cas9. Cell Stress Chaperones 23, 347–355. 10.1007/s12192-017-0845-2 28944418 PMC5904078

[B63] Park K. E. Park C. H. Powell A. Martin J. Donovan D. M. Telugu B. P. (2016). Targeted gene knockin in porcine somatic cells using CRISPR/Cas ribonucleoproteins. Int. J. Mol. Sci. 17:E810. 10.3390/ijms17060810 27240344 PMC4926344

[B64] Pattanayak V. Lin S. Guilinger J. P. Ma E. Doudna J. A. Liu D. R. (2013). High-throughput profiling of off-target DNA cleavage reveals RNA-programmed Cas9 nuclease specificity. Nat. Biotechnol. 31, 839–843. 10.1038/nbt.2673 23934178 PMC3782611

[B65] Peng Y. Clark K. J. Campbell J. M. Panetta M. R. Guo Y. Ekker S. C. (2014). Making designer mutants in model organisms. Development 141, 4042–4054. 10.1242/dev.102186 25336735 PMC4302887

[B66] Peterson B. A. Haak D. C. Nishimura M. T. Teixeira P. J. James S. R. Dangl J. L.. (2016). Genome-wide assessment of efficiency and specificity in CRISPR/Cas9 mediated multiple site targeting in Arabidopsis. PLoS ONE 11:e0162169. 10.1371/journal.pone.0162169 27622539 PMC5021288

[B67] Platt R. J. Chen S. Zhou Y. Yim M. J. Swiech L. Kempton H. R.. (2014). CRISPR-Cas9 knockin mice for genome editing and cancer modeling. Cell 159, 440–455. 10.1016/j.cell.2014.09.014 25263330 PMC4265475

[B68] Port F. Chen H. M. Lee T. Bullock S. L. (2014). Optimized CRISPR/Cas tools for efficient germline and somatic genome engineering in Drosophila. Proc. Natl. Acad. Sci. U.S.A. 111, E2967–E2976. 10.1073/pnas.1405500111 25002478 PMC4115528

[B69] Port F. Muschalik N. Bullock S. L. (2015). Systematic evaluation of *Drosophila* CRISPR tools reveals safe and robust alternatives to autonomous gene drives in basic research. G3 5, 1493–1502. 10.1534/g3.115.019083 25999583 PMC4502383

[B70] Qi L. S. Larson M. H. Gilbert L. A. Doudna J. A. Weissman J. S. Arkin A. P.. (2013). Repurposing CRISPR as an RNA-γuided platform for sequence-specific control of gene expression. Cell 152, 1173–1183. 10.1016/j.cell.2013.02.022 23452860 PMC3664290

[B71] Ran F. A. Hsu P. D. Lin C. Gootenberg J. S. Trevino A. Scott D. A.. (2014). Double nicking by RNA-guided CRISPR Cas9 for enhanced genome editing specificity. Cell 154, 1380–1389. 10.1016/j.cell.2013.08.021 23992846 PMC3856256

[B72] Rodgers K. McVey M. (2016). Error-prone repair of DNA double-strand breaks. J. Cell. Physiol. 231, 15–24. 10.1002/jcp.25053 26033759 PMC4586358

[B73] Rosenthal N. Brown S. (2007). The mouse ascending: perspectives for human-disease models. Nat. Cell Biol. 9, 993–999. 10.1038/ncb437 17762889

[B74] Ryder P. McHale M. Fort A. Spillane C. (2017). Generation of stable nulliplex autopolyploid lines of *Arabidopsis thaliana* using CRISPR/Cas9 genome editing. Plant Cell Rep. 36, 1005–1008. 10.1007/s00299-017-2125-0 28289885

[B75] Sakuma T. Nakade S. Sakane Y. Suzuki K. I. T. Yamamoto T. (2016). MMEJ-Assisted gene knock-in using TALENs and CRISPR-Cas9 with the PITCh systems. Nat. Protoc. 11, 118–133. 10.1038/nprot.2015.140 26678082

[B76] Shalem O. Sanjana E. N. Hartenian E. Zhang F. (2014). Genome-scale CRISPR-Cas9 knockout. Science 343, 84–88. 10.1126/science.1247005 24336571 PMC4089965

[B77] Shen Z. Zhang X. Chai Y. Zhu Z. Yi P. Feng G.. (2014). Conditional knockouts generated by engineered CRISPR-Cas9 endonuclease reveal the roles of coronin in *C. elegans* neural development. Dev. Cell 30, 625–636. 10.1016/j.devcel.2014.07.017 25155554

[B78] Shetty D. K. Inamdar M. S. (2016). Generation of a heterozygous knockout human embryonic stem cell line for the OCIAD1 locus using CRISPR/CAS9 mediated targeting: BJNhem20-OCIAD1-CRISPR-20. Stem Cell Res. 16, 207–209. 10.1016/j.scr.2015.12.041 27345969

[B79] Shi Z. Wang F. Cui Y. Liu Z. Guo X. Zhang Y.. (2015). Heritable CRISPR/Cas9-mediated targeted integration in Xenopus tropicalis. FASEB J. 29, 4914–4923. 10.1096/fj.15-273425 26268927

[B80] Shinmyo Y. Tanaka S. Tsunoda S. Hosomichi K. Tajima A. Kawasaki H. (2016). CRISPR/Cas9-mediated gene knockout in the mouse brain using in utero electroporation. Sci. Rep. 6, 1–13. 10.1038/srep20611 26857612 PMC4746659

[B81] Silva G. Poirot L. Galetto R. Smith J. Montoya G. Duchateau P.. (2011). Meganucleases and other tools for targeted genome engineering: perspectives and challenges for gene therapy. Curr. Gene Ther. 11, 11–27. 10.2174/156652311794520111 21182466 PMC3267165

[B82] Song B. Fan Y. He W. Zhu D. Niu X.. (2015). Improved hematopoietic differentiation efficiency of gene-corrected beta-thalassemia induced pluripotent stem cells by CRISPR/Cas9 system. Stem cells and development, 24, 1053–1065. 10.1089/scd.2014.0347 25517294

[B83] Tahara S. Nojima S. Ohshima K. Hori Y. Kurashige M. Wada N.. (2016). S100A4 accelerates the proliferation and invasion of endometrioid carcinoma and is associated with the “MELF” pattern. Cancer Sci. 107, 1345–1352. 10.1111/cas.12999 27348205 PMC5021035

[B84] Tsai S. Q. Wyvekens N. Khayter C. Foden J. A. Thapar V. Reyon D.. (2014). Dimeric CRISPR RNA-guided FokI nucleases for highly specific genome editing. Nat. Biotechnol. 32, 569–576. 10.1038/nbt.2908 24770325 PMC4090141

[B85] Tsarmpopoulos I. Gourgues G. Blanchard A. Vashee S. Jores J. Lartigue C.. (2016). In-yeast engineering of a bacterial genome using CRISPR/Cas9. ACS Synth. Biol. 5, 104–109. 10.1021/acssynbio.5b00196 26592087

[B86] Tsutsui H. Higashiyama T. (2017). PKAMA-ITACHI vectors for highly efficient CRISPR/Cas9-mediated gene knockout in *Arabidopsis thaliana*. Plant Cell Physiol. 58, 46–56. 10.1093/pcp/pcw191 27856772 PMC5444565

[B87] Van Sinay E. Mirabeau O. Depuydt G. Van Hiel M. B. Peymen K. Watteyne J.. (2017). Evolutionarily conserved TRH neuropeptide pathway regulates growth in *Caenorhabditis elegans*. Proc. Natl. Acad. Sci. U.S.A. 114, E4065–E4074. 10.1073/pnas.1617392114 28461507 PMC5441806

[B88] Wang H. La Russa M. Qi L. S. (2016). CRISPR/Cas9 in genome editing and beyond. Annu. Rev. Biochem. 85, 227–264. 10.1146/annurev-biochem-060815-014607 27145843 PMC13384723

[B89] Wang J. Song X. Guo C. Wang Y. Yin Y. (2016). Establishment of MAGEC2-knockout cells and functional investigation of MAGEC2 in tumor cells. Cancer Sci. 107, 1888–1897. 10.1111/cas.13082 27636589 PMC5198962

[B90] Wang L. Yang L. Guo Y. Du W. Yin Y. Zhang T.. (2017a). Enhancing targeted genomic DNA editing in chicken cells using the CRISPR/Cas9 system. PLoS ONE 12:e0169768. 10.1371/journal.pone.0169768 28068387 PMC5222187

[B91] Wang L. Yi F. Fu L. Yang J. Wang S. Wang Z.. (2017b). CRISPR/Cas9-mediated targeted gene correction in amyotrophic lateral sclerosis patient iPSCs. Protein Cell 8, 365–378. 10.1007/s13238-017-0397-3 28401346 PMC5413600

[B92] Wang M. Lu Y. Botella J. R. Mao Y. Hua K. Zhu J. K. (2017). Gene targeting by homology-directed repair in rice using a geminivirus-based CRISPR/Cas9 system. Mol. Plant 10, 1007–1010. 10.1016/j.molp.2017.03.002 28315751

[B93] Wang Y. Li Z. Xu J. Zeng B. Ling L. You L.. (2013). The CRISPR/Cas System mediates efficient genome engineering in *Bombyx mori*. Cell Res. 23, 1414–1416. 10.1038/cr.2013.146 24165890 PMC3847576

[B94] Wei W. Xin H. Roy B. Dai J. Miao Y. Gao G. (2014). Heritable genome editing with CRISPR/Cas9 in the silkworm, *Bombyx mori*. PLoS ONE 9:e101210. 10.1371/journal.pone.0101210 25013902 PMC4094479

[B95] Wills E. S. te Morsche R. H. M. van Reeuwijk J. Horn N. Geomini I. van de Laarschot L. F. M.. (2017). Liver cyst gene knockout in cholangiocytes inhibits cilium formation and Wnt signaling. Hum. Mol. Genet. 26, 4190–4202. 10.1093/hmg/ddx308 28973524

[B96] Xiong X. Zhang Y. Yan J. Jain S. Chee S. Ren B.. (2017). A scalable epitope tagging approach for high throughput ChIP-Seq analysis. ACS Synth. Biol. 6, 1034–1042. 10.1021/acssynbio.6b00358 28215080 PMC5536957

[B97] Xue Z. Ren M. Wu M. Dai J. Rong Y. S. Gao G. (2014). Efficient gene knock-out and knock-in with transgenic Cas9 in Drosophila. G3 4, 925–929. 10.1534/g3.114.010496 24657904 PMC4025491

[B98] Yang H. Wang H. Shivalila C. S. Cheng A. W. Shi L. Jaenisch R. (2013). XOne-step generation of mice carrying reporter and conditional alleles by CRISPR/cas-mediated genome engineering. Cell 154, 1370–1379. 10.1016/j.cell.2013.08.022 23992847 PMC3961003

[B99] Yin H. Kauffman K. J. Anderson D. G. (2017a). Delivery technologies for genome editing. Nat. Rev. Drug Discov. 16, 387–399. 10.1038/nrd.2016.280 28337020

[B100] Yin H. Song C. Dorkin J. R. Zhu L. J. Li Y. Wu Q.. (2017b). Therapequtic genome editing by combined viral and non-viral delivery of CRISPR system components *in vivo*. Nat. Biotechnol. 34, 328–333. 10.1038/nbt.3471 26829318 PMC5423356

[B101] Yin H. Song C. Q. Suresh S. Kwan S. Y. Wu Q. Walsh S.. (2018). Partial DNA-guided Cas9 enables genome editing with reduced off-target activity. Nat. Chem. Biol. 14, 311–316. 10.1038/nchembio.2559 29377001 PMC5902734

[B102] Yin H. Xue W. Chen S. Bogorad R. L. Benedetti E. Grompe M.. (2015). Genome editing with Cas9 in adult mice corrects a disease mutation and phenotype. Nat. Biotechnol. 32, 551–553. 10.1038/nbt.2884 24681508 PMC4157757

[B103] Yin L. Maddison L. A. Li M. Kara N. Lafave M. C. Varshney G. K.. (2015). Multiplex conditional mutagenesis using transgenic expression of Cas9 and sgRNAs. Genetics 200, 431–441. 10.1534/genetics.115.176917 25855067 PMC4492370

[B104] Yoshimi K. Kunihiro Y. Kaneko T. Nagahora H. Voigt B. Mashimo T. (2016). ssODN-mediated knock-in with CRISPR-Cas for large genomic regions in zygotes. Nat. Commun. 7:10431. 10.1038/ncomms10431 26786405 PMC4736110

[B105] Yu W. Mookherjee S. Chaitankar V. Hiriyanna S. Kim J.-W. Brooks M.. (2017). Nrl knockdown by AAV-delivered CRISPR/Cas9 prevents retinal degeneration in mice. Nat. Commun. 8:14716. 10.1038/ncomms14716 28291770 PMC5355895

[B106] Zetsche B. Gootenberg J. S. Abudayyeh O. O. Slaymaker I. M. Makarova K. S. Essletzbichler P. (2016). Cpf1 is a single-RNA-guided endonuclease of a Class 2 CRISPR-Cas system. Transgenic Res. 25, 207 10.1016/j.cell.2015.09.038 PMC4638220 26422227

[B107] Zhang L. Gao X. Wen J. Ning Y. Chen Y. G. (2006). Dapper 1 antagonizes Wnt signaling by promoting dishevelled degradation. J. Biol. Chem. 281, 8607–8612. 10.1074/jbc.M600274200 16446366

[B108] Zhang Y. Heidrich N. Ampattu B. J. Gunderson C. W. Seifert H. S. Schoen C.. (2013). Processing-independent CRISPR RNAs limit natural transformation in *Neisseria meningitidis*. Mol. Cell 50, 488–503. 10.1016/j.molcel.2013.05.001 23706818 PMC3694421

[B109] Zhang Y. Wang Y. Zuo Q. Li D. Zhang W. Wang F.. (2017). CRISPR/Cas9 mediated chicken Stra8 gene knockout and inhibition of male germ cell differentiation. PLoS ONE 12:e0172207. 10.1371/journal.pone.0172207 28234938 PMC5325261

[B110] Zhang Z. Aslam A. F. M. Liu X. Li M. Huang Y. Tan A. (2015). Functional analysis of Bombyx Wnt1 during embryogenesis using the CRISPR/Cas9 system. J. Insect Physiol. 79, 73–79. 10.1016/j.jinsphys.2015.06.004 26070541

[B111] Zhao P. Zhang Z. Ke H. Yue Y. Xue D. (2014). Oligonucleotide-based targeted gene editing in C. elegans via the CRISPR/Cas9 system. Cell Res. 24, 247–250. 10.1038/cr.2014.9 24418757 PMC3915902

[B112] Zuo E. Cai Y. J. Li K. Wei Y. Wang B. A. Sun Y.. (2017). One-step generation of complete gene knockout mice and monkeys by CRISPR/Cas9-mediated gene editing with multiple sgRNAs. Cell Res. 27, 933–945. 10.1038/cr.2017.81 28585534 PMC5518993

